# Supplementary material for: Blood transfusion service readiness and its associated factors in health facilities providing blood transfusion services across Ethiopia: A secondary analysis of the 2018 Service Availability and Readiness Assessment (SARA) survey
Source: PLoS One. 2024 Dec 12;19(12):e0315665. doi: 10.1371/journal.pone.0315665 (PMC11637254; doi:10.1371/journal.pone.0315665)
Supplement: Attachment — Submitted filename: Response to Reviewers.docx [file pone.0315665.s001.docx]

A point-by-point response to reviews

Dear Editor,

We, the authors, would like to thank the reviewers for their valuable comments which we feel have significantly strengthened our paper. Our point-by-point responses to the reviewers and editor are below each comment. We also reviewed to ensure that this version of the manuscript conforms to the journal style.

Response to Reviewers

Journal Requirements:

When submitting your revision, we need you to address these additional requirements.

1. Please ensure that your manuscript meets PLOS ONE's style requirements, including those for file naming. The PLOS ONE style templates can be found at

[https://journals.plos.org/plosone/s/file?id=wjVg/PLOSOne_formatting_sample_main_body.pdf](https://c05y1x9s.r.us-east-2.awstrack.me/L0/https:%2F%2Fjournals.plos.org%2Fplosone%2Fs%2Ffile%3Fid=wjVg%2FPLOSOne_formatting_sample_main_body.pdf/1/010f01920e9284fa-19c83484-b238-41b1-b3e3-283fc426d645-000000/a3qOUimFHu5OHss0WpmBAWUaZkg=177) and

[https://journals.plos.org/plosone/s/file?id=ba62/PLOSOne_formatting_sample_title_authors_affiliations.pdf](https://c05y1x9s.r.us-east-2.awstrack.me/L0/https:%2F%2Fjournals.plos.org%2Fplosone%2Fs%2Ffile%3Fid=ba62%2FPLOSOne_formatting_sample_title_authors_affiliations.pdf/1/010f01920e9284fa-19c83484-b238-41b1-b3e3-283fc426d645-000000/E1HhcoSThIqRFWSXgQvItcC05is=177)

Response: In this version, we have updated the figure in-text citations accordingly and ensured that the manuscript adheres to PLOS ONE's style requirements.

2. For studies involving third-party data, we encourage authors to share any data specific to their analyses that they can legally distribute. PLOS recognizes, however, that authors may be using third-party data they do not have the rights to share. When third-party data cannot be publicly shared, authors must provide all information necessary for interested researchers to apply to gain access to the data. ([https://journals.plos.org/plosone/s/data-availability#loc-acceptable-data-access-restrictions](https://c05y1x9s.r.us-east-2.awstrack.me/L0/https:%2F%2Fjournals.plos.org%2Fplosone%2Fs%2Fdata-availability%23loc-acceptable-data-access-restrictions/1/010f01920e9284fa-19c83484-b238-41b1-b3e3-283fc426d645-000000/SbwWs9HSNaUKIgR-IfuDeKJ6OfY=177))

For any third-party data that the authors cannot legally distribute, they should include the following information in their Data Availability Statement upon submission:

1) A description of the data set and the third-party source
2) If applicable, verification of permission to use the data set
3) Confirmation of whether the authors received any special privileges in accessing the data that other researchers would not have
4) All necessary contact information others would need to apply to gain access to the data

*Response: We updated the Data Availability Statement as follows:*

*Conducted by the Ethiopian Public Health Institute (EPHI) between October and December 2017, the SARA 2018 dataset—generated from a facility-based cross-sectional survey—offers insights into service availability and readiness for general healthcare and various specific services at both national and regional levels. Interested researchers can access the dataset by following a data-sharing agreement.*

*Researchers can request the dataset through EPHI’s data repository website for the “Ethiopia Services Availability and Readiness Assessment (SARA) Survey, 2018,” available at* [*https://rtds.ephi.gov.et/public/showdetail/94*](https://rtds.ephi.gov.et/public/showdetail/94)*. An assigned officer will review and facilitate the request. Upon submission, researchers will receive an email outlining the requirements, which include: 1) completing and returning the data request form; 2) providing a copy of the ethical approval certificate; and 3) attaching an official data request letter.*

3. Please upload a new copy of Figure 1 as the detail is not clear. Please follow the link for more information:

[https://blogs.plos.org/plos/2019/06/looking-good-tips-for-creating-your-plos-figures-graphics/](https://c05y1x9s.r.us-east-2.awstrack.me/L0/https:%2F%2Fblogs.plos.org%2Fplos%2F2019%2F06%2Flooking-good-tips-for-creating-your-plos-figures-graphics%2F/1/010f01920e9284fa-19c83484-b238-41b1-b3e3-283fc426d645-000000/4mY3knipz70hCQ0r0R26LY7YoF8=177)

[https://blogs.plos.org/plos/2019/06/looking-good-tips-for-creating-your-plos-figures-graphics/](https://c05y1x9s.r.us-east-2.awstrack.me/L0/https:%2F%2Fblogs.plos.org%2Fplos%2F2019%2F06%2Flooking-good-tips-for-creating-your-plos-figures-graphics%2F/2/010f01920e9284fa-19c83484-b238-41b1-b3e3-283fc426d645-000000/kCx9tAg678OVlbnzvbWUmdQx2pA=177)

Response: Figure 1 with improved resolution has been uploaded.

4. Please include a copy of Table 5 which you refer to in your text on page 13.

Response: It was a typo referring to Table 4, which has now been corrected.

5. Please review your reference list to ensure that it is complete and correct. If you have cited papers that have been retracted, please include the rationale for doing so in the manuscript text, or remove these references and replace them with relevant current references. Any changes to the reference list should be mentioned in the rebuttal letter that accompanies your revised manuscript. If you need to cite a retracted article, indicate the article’s retracted status in the References list and also include a citation and full reference for the retraction notice.

Response: Thank you. The references are checked and corrected for completeness.

**Additional Editor Comments:**

Please define the terms SSA and SARA as requested.
Please take note of the statistical terminology requested by reviewers.

Response: Thank you. The abbreviations have been defined, and all necessary changes have been made according to the reviewers' comments.


**Reviewers' comments:**

Reviewer #1: Thanks to the authors for this interesting manuscript. I have a few suggested edits/comments that you may wish to consider. Critically, it would be helpful to have a bit more methodological clarity and detail to enable a full interpretation of results.

Response: We well-acknowledged the valuable comments. In this version, we have addressed them and improved the manuscript accordingly.
Abstract
- What is meant by “determinants” of blood transfusion services? I think keeping it to readiness alone better reflects what was carried out.

Response: Thank you for the valid comments. Comment well taken and edited as follows “This study examined the readiness of blood transfusion services in health facilities across Ethiopia and its determinants.”

- Please swap commas for semi-colons between the components in lines 19–22.

Response: Comment well taken and edited accordingly.

Introduction
- In the first two sentences, there is no link made to the importance of blood transfusion for these conditions.
- It’s also not clear in lines 41/41 (over half of the deaths result from conditions that can be managed through prehospital and facility-based emergency care) what the reference is to needing blood. Please make this more explicit.

Response: The texts of the first two sentences as well as lines 41/42 have been revised for clarity as follows “Safe blood transfusion is essential for treating injuries and emergency obstetric conditions, serving as a life-saving intervention in various clinical scenarios. Timely and secure blood transfusion services are particularly vital in LMICs, where over half of deaths result from conditions manageable through emergency care.”

- Before you introduce what influences readiness, it would be worthwhile to define what you mean by readiness within the context of this study

Response: Thank you for the valid comments. The following definition is included in the text “Blood transfusion service readiness —defined as the preparedness and capability of a facility to provide safe, timely, and effective blood products and services, which includes trained staff, blood storage refrigerators, blood typing, cross-matching, supply safety and sufficiency, and guidelines for appropriate use—is crucial for ensuring safe and effective transfusions [12].” Lines 55-58

- Please define SSA (line 50)

- Remove “the” in line 58
- Please remove any acronyms that are not repeated in the manuscript (e.g. ERCS)

Response: We have defined the acronym SSA, removed 'the' where necessary, and eliminated the acronym ERCS accordingly.
Methods
- It’s not clear how many facilities participated at what level in your study. The 2018 SARA included 764 facilities, a census of 303 hospitals, 164 health centers, 165 clinics, **excluding** the health posts, but you note that you included 632 facilities, excluding health posts, participated in your study? Why not all of the facilities in the SARA? It would also be helpful to see what those 632 facilities were in terms of level. Please clarify what the overall sample size (and if there was a calculation, please provide this) was and how it was expected to be generalizable (i.e. what parameters you used for sample size calculations to ensure this, especially as regressions were used—how did you ensure you had sufficient numbers for each variable to do this type of analysis?).
- To that end, a table would be enormously useful (e.g. with a column for total health facilities by region/type, a column for those included in the SARA, and then a column for those included in this analysis).

Response: The 2018 SARA included 632 hospitals, health centers, and clinics, along with 162 health posts. We included all 632 facilities (excluding the 132 health posts, which are not expected to provide blood transfusion services). Tables 1 and 2 present the facility levels and location. Regarding the adequacy of the sample size check, we have added the following sentences “In this study, we included all 632 facilities (except the health posts which are expected to provide blood transfusion services) in the SARA survey were included. The adequacy of the sample size is assessed using a single proportion formula with a 5% margin of error, a 95% confidence level, a design effect of 1.5, and an assumption that 88% of hospitals reported performing compatibility testing for whole blood and RBC [20]. Additionally, a double population comparison is conducted for blood transfusion service readiness, considering managing authority and facility type as exposure variables, with an assumed power of 80%, a design effect of 1.5, and a 95% confidence interval [16].” Lines 104-109

- Please remove “were” in line 96
- Please add “and” before “service readiness” in line 113

Response: We have removed the word 'were' and added 'and' accordingly.

- Interviews and a facility inventory questionnaire were carried out for the SARA, but it’s not clear what the function of the interviews was in terms of readiness? Which instrument actually gave insights around the seven tracer items used in the present study?

Response: Thank you for the comments. The following sentence has been added in lines 120-122. “Specific to facility readiness, the interviewer was instructed to interview the person most knowledgeable about blood transfusion services at the facility where blood is collected, processed, tested, stored, or handled prior to transfusion.”

- Please describe the seven tracer items in more detail, in terms of what was actually assessed for each, the time frames in which they may have been evaluated (e.g. previous three months? Only the day of the assessment

Response: Thanks for the valid comments. The table below has been added to clarify each tracer items detailing what it means including its time timeframe. Lines 128-139

| Items | Definitions and data sources |
| --- | --- |
| Guidelines on the appropriate use of blood and safe blood transfusion | Observed availability of guidelines on the appropriate use of blood and safe blood transfusion in the service area |
| Staff trained in the appropriate use of blood and safe blood transfusion | At least one staff member providing the service trained in the appropriate use of blood and safe blood transfusion within the past 2 years based on the interview response from in-charge of service area day of the survey |
| Blood storage refrigerator | Observed availability and reported functionality and with temperature being monitored (checked that temperature has been monitored at least once in the past 24 hours and maintained at 2 – 6 ^o^C) in the service area or adjacent area |
| Blood typing | Observed ability of the facility to conduct the ABO blood group test, Rhesus blood group test, and centrifuge tests on-site (in the facility) and functioning equipment and reagents needed to conduct the test on the day of the survey. |
| Cross-match testing | Observed ability of the facility to conduct the crossmatch (should use methods that demonstrate ABO incompatibility and incompatibility due to other clinically significant antibodies and should include an indirect anti-globulin test or a test of equivalent sensitivity), centrifuge, 37°C incubator, and grouping sera on-site on the day of the survey. |
| Blood supply sufficiency | Reported availability of no interruption of blood availability in the last three months |
| Blood supply safety | Reported availability of blood obtained only from national or regional blood bank, OR blood obtained from other sources but screened for HIV, Syphilis, Hepatitis B, and Hepatitis C |

Results
- Please add a “totals” row in Table 1.
- The chi-squared test is not mentioned in your methods. Further, it’s not clear to me why this was done at all? For instance, would you expect all facilities to provide blood transfusion? In Table 2, please ensure n/N is reflected. It’s not clear what the total facilities is (and further, is this ALL facilities in the region, or only those expected to carry out blood transfusion? If it’s the latter, the chi-squared is useful, but if it’s the former, I would not include this analysis).

Response: The total number of facilities expected to provide blood transfusion services are 632 sampled facilities in the country in the SARA survey. The N and n/N are included in Tables 1 and 2, respectively. Ch2 is mentioned in the analysis section. Lines 147-149.

- This is a minor comment, but please swap the [ ] and ( ) brackets (e.g. (Coef.: -0.74; 95% CI: 0.014 [-1.32--0.15]). In general, confidence intervals are usually in square brackets.

Discussion
- In line 192, please change “was significantly varied” to “varied significantly”.

- Please revise “the readiness in facilities providing” in line 201 to “the readiness of facilities to provide”.

Response: Thank you for bringing all these to our attention. These have been edited accordingly.

- A limitation of this study is that it relies on the SARA, which only allowed for “available” or “not available” , but I imagine in many facilities (as has been my own experience), there is availability “sometimes”. How were these facilities coded? If it had to be all the time to be coded “available”, then the overall picture presented would be quite conservative, reflecting a worse picture. If there just had to be some availability for “available”, then the converse is true. It would be useful for the authors to reflect on the implications of this coding for reflecting the overall state of blood transfusion services.

Response: This is an interesting insight. The survey estimates the all-time availability of blood transfusion services, which is indeed a concerning situation as you noted. We have reflected this in the Discussion section, lines 226-228. *“As the SARA survey provided a conservative estimate of the all-time availability of blood transfusion services, highlighting a concerning situation that would significantly impact access to emergency care. “*

- It also strikes me as a clear finding that only 256 facilities actually provide blood transfusion, in practice, as I imagine the number, in theory is much higher. In your discussion you might consider adding a reflection on the proportion of facilities expected to offer blood transfusion services that actually do and the implications of this.
- It would also be useful to see some reflections by the authors on the implications of this study for both policy and for practice.

Response: Comment well taken. We have discussed this dedicating a paragraph Lines 223-230

“The study revealed that a significant number of hospitals and most health centers and clinics lacked blood transfusion services, despite the expectation that hospitals and certain upgraded health centers and clinics should offer these services—particularly for emergencies, maternal health care, and other critical care situations. As the SARA survey provided a conservative estimate of the all-time availability of blood transfusion services, highlighting a concerning situation that would significantly impact access to emergency care. Previous studies have also shown that limited access, primarily due to the physical distance to facilities, is a major factor contributing to delays in receiving necessary care [21-23].”

- Please revise “is” to “were” in line 235, and “investments” to “investing”, “in availing” to “to avail”, and “addressing” to “to address” in line 237.
- In the list of acronyms, it should be “odds ratio”, not “odd ratio”

Response: Thank you again for bringing them to our attention. We have edited them accordingly.

Reviewer #2: A research study was conducted which aimed to examine the readiness and determinants of blood transfusion services in Ethiopian facilities.
Minor revisions:
1- The standard statistical term for average is mean. When providing means, also state the corresponding standard deviations.
2- Line 127: To improve clarity consider replacing “for” to “with”.
3- Line 136: Add “for” to “check for multicollinearity”.

Response: Thank you for bringing them to our attention. We have edited them accordingly.

4- P-values never equal zero; express small p-values as < 0.001.
5- Line 169: State which pairwise regions and facility types differed with respect to mean readiness scores.

Response: Thank you for the valid comments. In this version, we presented pairwise comparisons between regions and facility types and described this in the Analysis and Results sections as follows.

Furthermore, pairwise comparisons between regions and facility types were conducted, adjusting for multiple comparisons using the Bonferroni correction.

Pairwise comparisons revealed significant differences in the mean readiness scores between the following groups: Addis Ababa and the Somali region; specialized hospitals and primary hospitals; specialized hospitals and health centers and clinics; as well as general hospitals and health centers and clinics.

6- Line 128: The Student’s t-test is appropriate for comparing two groups. Locations and facility types have more than two groups, correct? ANOVA is an extension of the Student's t-test for comparing more than two groups.

Response: It has been edited accordingly. Thanks.

Reviewer #3: Methods
- Measurement: I am not clear on how you obtain the mean percentage service readiness score. The description seemed confusing to me. What are the cut-off points?

Response: Tracer the items included for measuring readiness and their definitions are included as Table 1 as asked by Review I to make clear that each item is measured as available or not available.

Results
- Please do not represent # as number. It is not a standard symbol in journals
- Line 152, first sentence is a repetition from your methods. I suggest rewording them
- p-value of 0.000 should be changed to <0.001
- Line 179: Table 5 does not exist

Response: Thank you for bringing them to our attention. We have edited them accordingly.

- Please change the way you present your regression analysis. Your p-value and 95%CI are reversed

Response: Comment well taken and 95% CI and p-values are presented in separate columns

Discussion
- How do you determine your results to be sub-optimal or optimal? Refer to methods comment.

Response: Thank you. We have presented the details of each tracer item and their definitions in the methods section.

- We need more comparisons with other studies. If not African countries, how about other low-middle income countries for comparison?
- Discussions for the availability of medical equipment is lacking. Please expand on your findings.
Response: Thank you for the valid comment. Though there is limited studies on blood transfusion readiness of facilities, we tried our best to expand the Discussion.

Miscellaneous
- Please spell out the full term at its first mention, indicate its abbreviation in parenthesis and use the abbreviation from then on (SSA, SARA)

Response: Now addressed accordingly, thanks.
