# Supplementary material for: Blood transfusion service readiness and its associated factors in health facilities providing blood transfusion services across Ethiopia: A secondary analysis of the 2018 Service Availability and Readiness Assessment (SARA) survey
Source: PLoS One. 2024 Dec 12;19(12):e0315665. doi: 10.1371/journal.pone.0315665 (PMC11637254; doi:10.1371/journal.pone.0315665)
Supplement: Attachment — Submitted filename: Reviewer comments_PONE-D-24-30780R1.docx [file pone.0315665.s002.docx]

Reviewer 1

All comments have been addressed. Many thanks to the authors for their revised manuscript. This is reading more clearly now and I'm sure will make a useful contribution to literature.

Reviewer 2

Minor revision:

Line 104: For the sample size estimate, indicate the statistical testing method used to generate the confidence interval.

Reviewer 3

Thank you for addressing all the reviewers' comments in your manuscript. I still have one issue with the writing that has not been resolved.

In your methods, you have clearly outlined the seven tracer items and their definitions, and mentioned how you obtain the mean percentage service readiness score. Now, how do you determine what is the cut-off mean score?

Situation 1:

If 7 is the maximum score, would a mean score of 3.5 (50%) be the cut-off point between being optimum (>50%) and suboptimum (<50%)?

Situation 2:

A 70% a cut-off point, according to other SARA studies I've found.

Situation 3:

You simply categorize any score <100% a suboptimal score, according to what you have described in your discussion. In table 4, all mean scores according to the region, facility, managing authority and residence is at the range of 50% - 72.4%. Therefore, the service readiness is at "suboptimal" level?

Whether the mean score readiness is set arbitrarily, or at a standard, or otherwise, please mention it in your methods.
