# Supplementary material for: Blood transfusion service readiness and its associated factors in health facilities providing blood transfusion services across Ethiopia: A secondary analysis of the 2018 Service Availability and Readiness Assessment (SARA) survey
Source: PLoS One. 2024 Dec 12;19(12):e0315665. doi: 10.1371/journal.pone.0315665 (PMC11637254; doi:10.1371/journal.pone.0315665)
Supplement: Attachment — Submitted filename: Response to Reviewers.docx [file pone.0315665.s003.docx]

A point-by-point response to reviews

Dear Alemu Birara,

Academic Editor,

We, the authors, would like to express our gratitude to the reviewers and editors of PLOS ONE Journal for their valuable comments, which we believe have significantly strengthened our paper. Below, we provide point-by-point responses to each comment and suggestions in red font. We have also reviewed the manuscript to ensure that it conforms to the journal's style.

**Journal Requirements**

Please review your reference list to ensure that it is complete and correct. If you have cited papers that have been retracted, please include the rationale for doing so in the manuscript text, or remove these references and replace them with relevant current references. Any changes to the reference list should be mentioned in the rebuttal letter that accompanies your revised manuscript. If you need to cite a retracted article, indicate the article’s retracted status in the References list and also include a citation and full reference for the retraction notice.

Response: The reference lists have been reviewed, and minor corrections have been made to the format

**Reviewers' comments**

Reviewer 1

All comments have been addressed. Many thanks to the authors for their revised manuscript. This is reading more clearly now and I'm sure will make a useful contribution to literature.

Response: Thank you for taking the time to review our manuscript.

Reviewer 2

Minor revision:

Line 104: For the sample size estimate, indicate the statistical testing method used to generate the confidence interval.

Response: Thank you for bringing this to our attention. We used the Wilson score interval method to generate the confidence interval for the proportion estimate and to assess the adequacy of the sample size. The following sentence has been included in the revised *version “The Wilson score interval method was used to generate the confidence interval for the proportion estimate."* See the Revised Manuscript with Track Changes, page 7, lines 105–106.

Thank you for your valuable feedback.

Reviewer 3

Thank you for addressing all the reviewers' comments in your manuscript. I still have one issue with the writing that has not been resolved.

In your methods, you have clearly outlined the seven tracer items and their definitions, and mentioned how you obtain the mean percentage service readiness score. Now, how do you determine what is the cut-off mean score?

Situation 1:

If 7 is the maximum score, would a mean score of 3.5 (50%) be the cut-off point between being optimum (>50%) and suboptimum (<50%)?

Situation 2:

A 70% a cut-off point, according to other SARA studies I've found.

Situation 3:

You simply categorize any score <100% a suboptimal score, according to what you have described in your discussion. In table 4, all mean scores according to the region, facility, managing authority and residence is at the range of 50% - 72.4%. Therefore, the service readiness is at "suboptimal" level?

Whether the mean score readiness is set arbitrarily, or at a standard, or otherwise, please mention it in your methods.

Response: Thank you for your insightful explanation and guidance. We sincerely apologize for overlooking your comments in the previous version. Initially, we believed we had used the mean readiness score as a continuous variable. However, as you rightly pointed out, we categorized it into suboptimal and optimal scores in our Discussion section, while also reporting the mean score of 65% in the Results section.

Accordingly, we have applied the 70% cut-off point, as you suggested, and as is commonly used in previous SARA-based surveys for assessing readiness in chronic disease services. The following sentence has been included in the revised version, with the corresponding references cited: *"A cut-off mean score of 70% or more is considered optimal for determining readiness to provide blood transfusion services [21, 22].",* Revised Manuscript with Track Changes, page 9, lines 142–143.

Thank you for your thorough review and guidance.
